# Functionalization Effect on Polymer Nanocomposite Coatings Based on TiO_2_–SiO_2_ Nanoparticles with Superhydrophilic Properties

**DOI:** 10.3390/nano8060369

**Published:** 2018-05-25

**Authors:** Arturo Román Vázquez-Velázquez, Miguel Angel Velasco-Soto, Sergio Alfonso Pérez-García, Liliana Licea-Jiménez

**Affiliations:** Centro de Investigación en Materiales Avanzados S.C., Unidad Monterrey, Alianza Norte No. 202, Parque PIIT, 66628 Apodaca, Nuevo León, Mexico; arturo.vazquez@cimav.edu.mx (A.R.V.-V.); miguel.velasco@cimav.edu.mx (M.A.V.-S.)

**Keywords:** TiO_2_–SiO_2_ functionalization, superhydrophilic coating, nanocomposite coating

## Abstract

In this study, a novel binary nanocomposite system based on TiO_2_-SiO_2_ was functionalized with trimethylolpropane triacrylate (TMPTA) and characterized by XPS and XRD. Results revealed that TiO_2_-SiO_2_ nanoparticles were covalently functionalized. Functionalized nanoparticles at low concentrations (0.1 wt % and 0.5 wt %) were dispersed in acrylic acid acting as a polymer matrix. Nanocomposite coatings analysis demonstrate to achieve superhydrophilic properties as well as very good optical characteristics. Water contact angle characterization showed the functionalization effect by achieving a superhydrophilic behavior with a contact angle less than 5°. UV-Vis measurements demonstrated high optical transmittance above 95% for the coatings. Based on the obtained results a mechanism describing the chemical interactions of the constituents responsible for the synergy in the nanocomposite as well as the morphological play role in the behavior are presented.

## 1. Introduction

New materials with novel properties are always required in order to overcome increasing technological demands. Some of these materials are based on polymer nanocomposites, in which dispersed nanoparticles are incorporated into a matrix. In order to improve the affinity of these phases and avoid agglomeration, surface modification is required on the nanoparticles; this process is known as functionalization.

It is known that the functionalization of nanoparticles has interesting advantages: the functional groups at the nanoparticle act as anchor points to other groups or molecules in the matrix, and it is possible to increase affinity with the receptor compound, for example, a polymeric matrix [1,2]. Through nanoparticles, functionalization makes it possible to obtain materials with functional features.

Nowadays, the formulation of polymer–inorganic nanocomposite materials has been widely researched because of their interesting properties; they combine the features of organic and inorganic materials, generating new materials for different potential applications. These materials have the synergy of the flexibility and processability of the polymers with the optical and hydrophilic properties of the inorganic component, generating a compatible compound with both features [3].

Recently, oxide nanoparticles have been studied as a reinforcement of polymer nanocomposites for many applications—for example, in the development of functional coatings with special properties, such as protective or self-cleaning features [4,5]. A variety of inorganic nanoparticles have been used for the development of self-cleaning coatings, mainly metal oxides such as TiO_2_ and SiO_2_. In addition, some self-cleaning coatings are known for showing superhydrophilicity, meaning droplet water contact angles are less than 5°.

Nanoparticles of TiO_2_ and SiO_2_ show interesting properties, such as optical, superhydrophilic, and photocatalytic features; thus these oxides can be considered as suitable materials for the development of self-cleaning and anti-reflective coatings [6,7,8] and for outdoor applications such as the building industry and solar panels, among others.

There exist several studies related to TiO_2_–SiO_2_ as films, where the effect of TiO_2_ addition to SiO_2_ on the optical, mechanical, wettability, and photocatalytic activity has been investigated [9,10,11,12,13]. Other studies have been carried out on TiO_2_–SiO_2_ mixing by the sol–gel method, where in some cases optical properties, particularly antireflection, have been compromised [5]. However, the preparation of polymer nanocomposite coatings based on TiO_2_–SiO_2_ has not yet been reported.

In order to achieve a polymer nanocomposite, surface modification of nanoparticles is required; typical functionalization agents include silanes [14], amines [15], and thiols [16], and modification conditions require low humidity and an inert atmosphere. Polyacrylic polymers exhibit good transparency, are hydrophilic, and can be a good candidate for coatings for which transparency is required. Trimethylolpropane triacrylate (TMPTA) is a molecule with a high degree of oxygen functionality and could be used as a crosslinking or functionalizing agent of metal oxide nanoparticles. Although related work exists, it is scarce [17,18] and without explanation of the chemical interactions.

As mentioned before, the goal of a nanocomposite is to achieve synergy of the properties of the polymer and the dispersed nanoparticles. One of the main effects for the nanoparticle functionalization is to increase the affinity with its matrix without altering the properties of the nanoparticle. In order to achieve functional nanocomposite coating properties such as self-cleaning, a critical step is to understand and control the interactions of functionalized nanoparticles in a polymeric matrix.

Here, a study of the functionalization effect on nanocomposite coatings with hydrophilic and superhydrophilic properties is presented, with results obtained through the surface modification of TiO_2_–SiO_2_ nanoparticles with TMPTA.

## 2. Materials and Methods

### 2.1. Materials

TiO_2_ nanoparticles (TiO_2_ P90) with a particle size of about 25 nm, a density of 120 g/L, and a specific surface of 90 ± 20 m^2^/g and SiO_2_ (Aerosil 200) with an average nanoparticle size of 12 nm, a density of 50 g/L, and a specific surface of 200 ± 25 m^2^/g were provided from Evonik. TMPTA of chemical grade inhibited with 600 ppm of monomethyl ether hydroquinone and an acrylic acid (AA) monomer (99%, inhibited with 200 ppm monomethyl ether hydroquinone) were purchased from Sigma-Aldrich, and ethanol was purchased from Fermont. The reagents were used as received.

### 2.2. Functionalization of TiO_2_–SiO_2_ Nanoparticles

Functionalization was carried out in a TiO_2_–SiO_2_ system with a 1:1 (*w*/*w*) ratio. The nanoparticle ratio was determined through previous experiments. First, nanoparticles were dispersed in 10 mL of ethanol with the aid of ultrasonication for 30 min using 100% amplitude, supplying 59,400 J of energy. Next, TMPTA was added to the previous nanoparticle dispersion in a 1:5 (*w*/*w*) ratio, and the solution was stirred vigorously for 4 h. Thereafter, the solution was sonicated for 2 h; the resultant solution was filtered through a polytetrafluoroethylene membrane filter with a pore size of about 0.2 µm and dried at 65 °C for 24 h. In Table 1, the nomenclature of the modified nanoparticles is shown, where f-TiO_2_–SiO_2_ refers to funtionalized nanoparticles.

### 2.3. Preparation of TiO_2_–SiO_2_ Embedded Nanocomposite Coatings

Solutions of non-functionalized and functionalized nanoparticles at 0.1 and 0.5 wt % in ethanol and AA (1:1 volume ratio) as the monomer were sonicated for 30 min, being supplied 594 kJ/g of energy and 100% amplitude. The obtained nanocomposites were used for film fabrication by spin coating; a wet rotational speed of 2000 rpm and a holding time of 9 s followed by a drying rotational speed of 2500 rpm and a holding time of 20 s were used. Glass slides (6.25 cm^2^) used as substrates were washed by sonication in cyclohexanone, chloroform, acetone, isopropanol, and deionized water for 15 min each. The obtained coatings were air-dried at 60 °C for 30 min. Next, the coatings were cured in the ultraviolet (UV) oven for 15 min. The nanocomposite coatings obtained presented a thickness of ~70 nm. The pure AA was used as a control to elucidate the effect of the nanoparticles’ addition. Samples were labeled as is shown in Table 1, where PAA refers to polyacrylic acid.

### 2.4. Characterization

In order to evaluate the nanoparticles’ surface modification, chemical state analysis was carried out by X-ray photoelectron spectroscopy (XPS) measurements using a Thermo Scientific Escalab 250 Xi instrument; calibration was done with sputtered gold (Au4f), silver (Ag3d), and copper (Cu3p) standards and the C1s aligned to 284.6 eV. The base pressure during the analysis was 10^−10^ mbar, and photoelectrons were generated by an Alkα (1486.68 eV) X-ray source equipped with a monochromator and with a spot size of 650 μm. The X-ray voltage and power were 14 kV and 350 W, respectively. The acquisition conditions for the low-resolution analysis for the survey were as follows: pass energy of 46.95 eV at a take-off angle of 45° and a 1 eV/step. For the high-resolution spectra within the selected regions, the conditions were as follows: 20 eV pass energy, 45° take-off angle, and 0.1 eV/step. Selected region spectra were recorded covering the Ti2p_3/2_, Ti2p_1/2_, Si2p_3/2_, and Si2p_1/2_ as well as C1s and O1s photoelectron peaks. The experimental error value was smaller than the binding energy shift. The recorded photoelectron peaks were finally fitted using Avantage software, version 5.41.

X-ray diffraction (XRD) was used to identify the structure of the nanoparticle powder before and after functionalization. The XRD pattern was recorded using an X’Pert X-ray diffractometer (Panalytical) in a 2*θ* range of 5–100° with a step size of 0.0170° and a scan rate of 1° min^−1^ using CuKα radiation. The water contact angles were measured with an OCA 15 plus (DataPhysics model) at room temperature in air following the ASTM5725-99 standard; SCA20 software was used for the analysis. Transmission spectra of the nanocomposite coatings under normally incident light were recorded on a Cary 5000 UV-Vis-NIR spectrophotometer (Agilent Technologies, México City, México). Morphologies of the nanocomposite coatings were analyzed by atomic force microscopy (AFM) (model MFP3D-SA, Asylum Research) using a rectangular cantilever with a resonant frequency of 70 kHz and a nominal force constant of 2 N/m; the topography images (1 µm × 1 µm) were taken in contact mode for each surface. Roughness calculations and three-dimensional (3D) images were obtained using ARgyle Light analysis software. Scanning electron microscope (SEM) images for the nanocomposite coatings deposited on glass were recorded using a FEI NovaNano SEM 2000 without gold coating with a helix detector in immersion mode, using 15 kV at 10,000× magnification for the 0.1% nanocomposite and 10 kV at 10,000× magnification for the 0.5% nanocomposite.

## 3. Results

### 3.1. Functionalization of TiO_2_–SiO_2_ Nanoparticles

**Figure 1:**
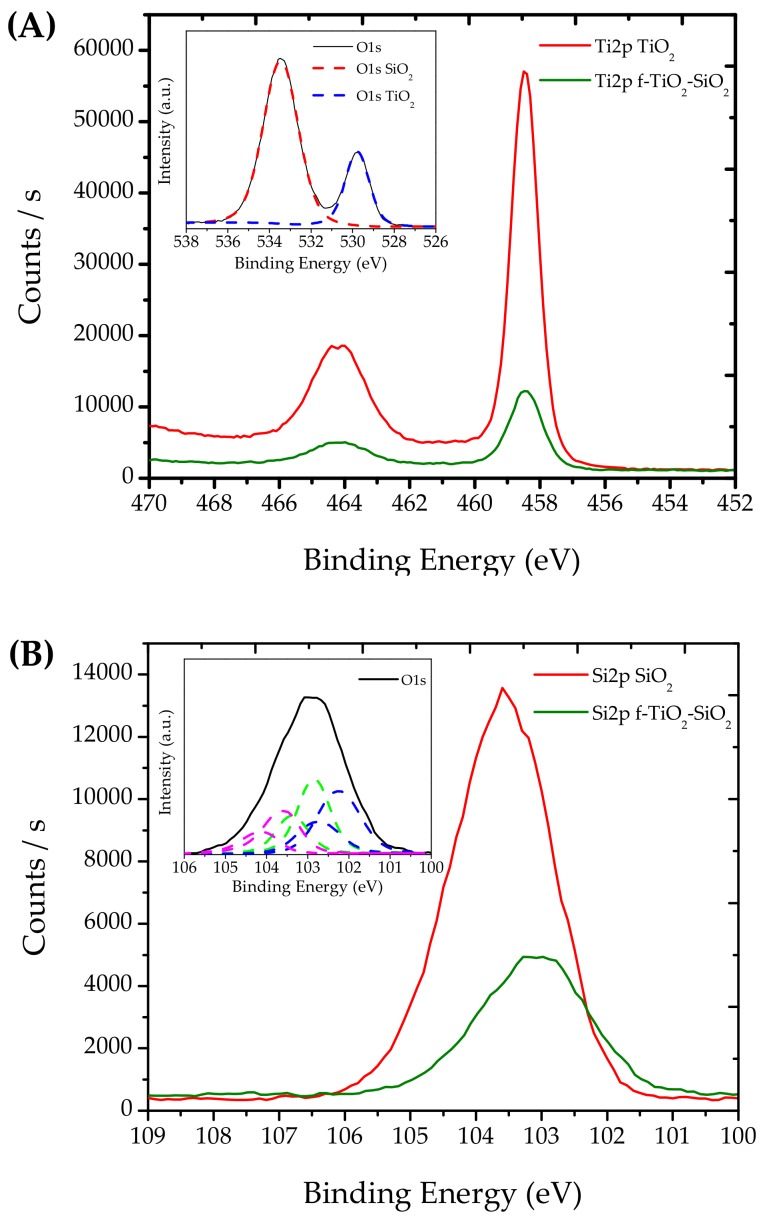
High-resolution X-ray photoelectron spectroscopy (XPS) spectra of (**A**) Ti2p for the pristine and functionalized nanoparticles and O1s (inset), (**B**) Si2p XPS spectra of the pristine and functionalized nanoparticles.

To understand the functionalization effect and the interaction between the TiO_2_ and SiO_2_ nanoparticles with TMPTA, the systems were analyzed by means of XPS. As shown in Figure 1, the Ti2p spectra (Figure 1A) of TiO_2_ showed the Ti2p_3/2_ signal with a binding energy at 458.5 eV and a Ti2p_3/2_–Ti2p_1/2_ separation of 5.74 eV; these spectra were in agreement with those for TiO_2_ pristine nanoparticles [19]. The Ti2p spectra for f-TiO_2_–SiO_2_ decreased in intensity and became broader; the Ti2p_3/2_ full width at half maximum (FWHM) increased from 1 to 1.23 eV, and the peak shape changed for the functionalization effect. The mathematical analysis of the signal corresponding to O1s of the f-TiO_2_–SiO_2_ sample could be adjusted with two different signals (Figure 1A inset), the first at 529.8 eV, and the second at 533.5 eV. The signal at 529.8 eV corresponded to O1s of TiO_2_ nanoparticles, and the signal at 533.5 eV was attributed to oxygen of the SiO_2_ nanoparticles. Furthermore, the Si2p spectra (Figure 1B) showed a signal at a binding energy of 103.6 eV; as did the Si2p spectra of nanoparticles obtained after functionalization, for which a broadening of the signal was observed, changing the FWHM from 1.93 to 2.14 eV as well as changing the peak position and shape. The peak could be analyzed using three doublet signals (Figure 1B inset), one at 102.3 and 102.7 eV for Si–Ti bonding, another at 102.9 and 103.4 eV for Si–C bonding, and another at 103.6 and 104.1 eV that was attributed to Si–O [20]. From this, we can infer that functionalization took place on the nanoparticle surface.

**Figure 2:**
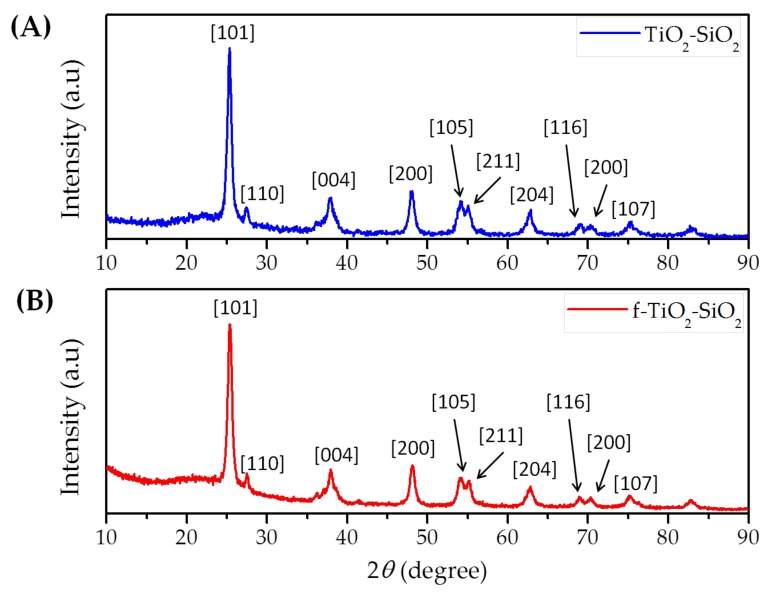
X-ray diffractograms of (**A**) TiO_2_–SiO_2_ and (**B**) f-TiO_2_–SiO_2_ nanoparticles.

To determinate that the nanoparticle structure remained after the functionalization process, XRD analysis was carried out. Figure 2 shows the X-ray diffractograms of the non-functionalized (Figure 2A) and functionalized nanoparticles (Figure 2B). The XRD patterns of the TiO_2_–SiO_2_ and f-TiO_2_–SiO_2_ samples demonstrated the presence of the anatase phase originated by TiO_2_, as well as a minor proportion of rutile [21,22] and an amorphous halo around 23°, which could be assigned to amorphous SiO_2_ [23,24]. These results suggest that the nanoparticles did not change their crystalline arrangement as a result of the functionalization process; thus it is possible to establish that functionalization only modified the nanoparticles surface.

XPS analysis confirmed that TMPTA effectively modified the surface of the TiO_2_ and SiO_2_ nanoparticles. Additionally, XRD diffractograms showed that no structural modifications were suffered during functionalization.

### 3.2. Surface Wettability of Nanocomposite Coatings

**Figure 3:**
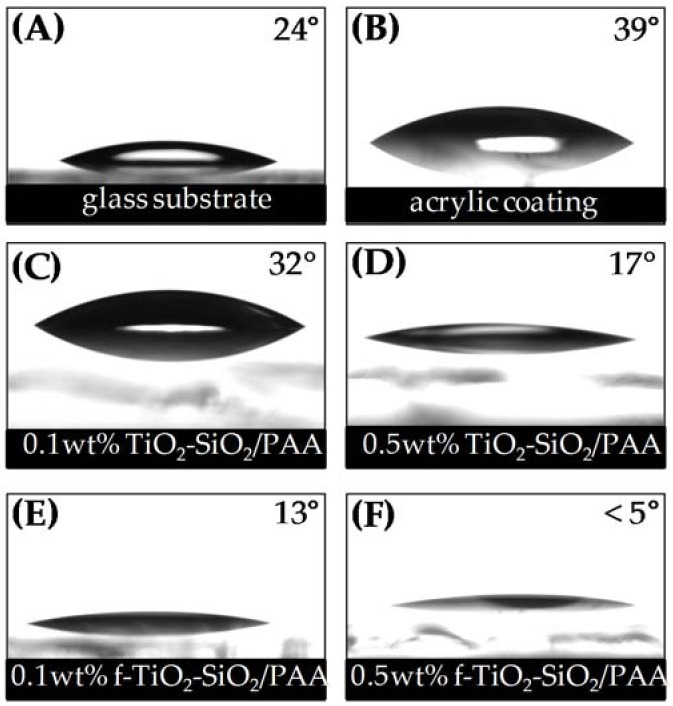
Images of water contact angle of (**A**) glass substrate, (**B**) acrylic coating, (**C**) 0.1 wt % TiO_2_–SiO_2_/PAA, (**D**) 0.5 wt % TiO_2_–SiO_2_/PAA, (**E**) 0.1 wt % f-TiO_2_–SiO_2_ /PAA, and (**F**) 0.5 wt % f-TiO_2_–SiO_2_/PAA.

Surface wettability is critical for self-cleaning applications. Figure 3 shows the wettability behavior of all evaluated coatings. The water contact angles on the glass substrate (Figure 3A) and polyacrylic coating (Figure 3B) were 24° (±1°) and 39° (±2°), respectively. The incorporation of TiO_2_–SiO_2_ nanoparticles decreased the water contact angle, as can be seen in Figure 3C–F. The results indicate that nanocomposites based on functionalized nanoparticles present smaller contact angles. At a nanoparticle concentration of 0.5 wt % (Figure 3F), the water contact angle was even smaller than that of the 0.1 wt % coating (Figure 3E). Evidently, the nanoparticle concentration is an important factor in the development of surfaces with hydrophilic properties; superhydrophilic behavior with less than a 5° water contact angle was observed for the case of the 0.5 wt % f-TiO_2_–SiO_2_/PAA sample. On the other hand, the non-functionalized nanoparticle coatings (Figure 3C,D) showed water contact angles of 32° (±2°) and 17° (±2°), respectively. It has been seen that the nanoparticle concentration and roughness (as shown below) are not the only factors that influence the wettability of nanocomposites; it is thought that wettability is determined by the interaction between the arrangement of functionalized nanoparticles and the polymer matrix. It can be remarked that the incorporation of modified nanoparticles to a polymer matrix produces nanocomposites with different features, a small water contact angle and superhydrophilic properties. These properties are strongly related to the chemical interactions between all the constituents in the nanocomposite.

### 3.3. Transparency of Nanocomposite Coatings

**Figure 4:**
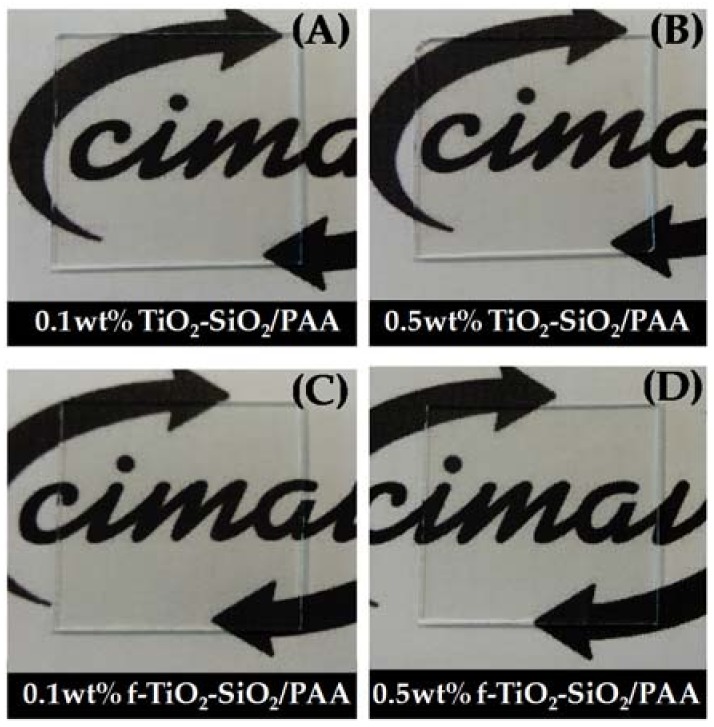
Images of nanocomposite coatings on glass substrates of (**A**) 0.1 wt % TiO_2_–SiO_2_/PAA, (**B**) 0.5 wt %TiO_2_–SiO_2_/PAA, (**C**) 0.1 wt % f-TiO_2_–SiO_2_/PAA, and (**D**) 0.5 wt % f-TiO_2_–SiO_2_/PAA.

Figure 4 depicts the captions of the nanocomposites coatings. As can be seen in the images, all coatings were highly transparent and homogeneous, lacking the presence of agglomerates on the surface.

**Figure 5:**
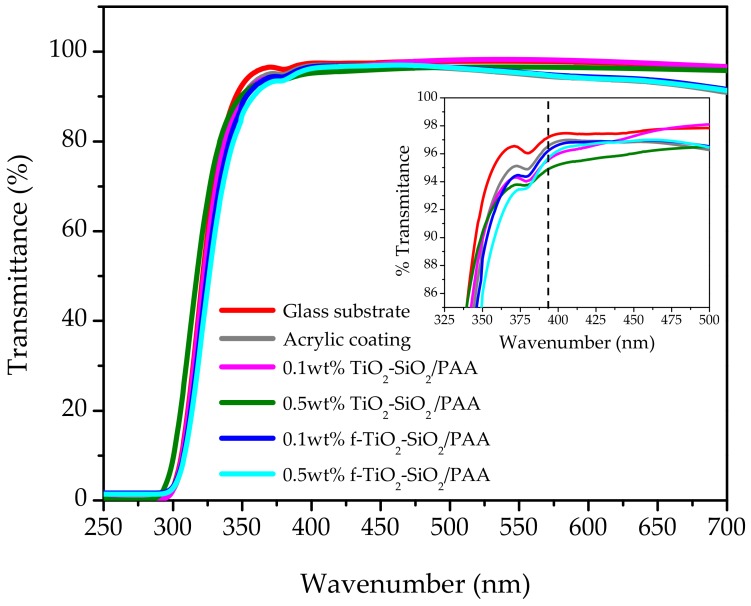
Transmission spectra of the prepared nanocomposite coatings at a wavelength ranging from 250 to 700 nm.

Transmission spectra of the nanocomposite coatings are shown in Figure 5. For comparison, the transmission spectra of a glass substrate and polyacrylic acid on glass are also shown. The glass substrate transmittance in the wavelength range between 370 and 700 nm was about 97.5%. Considering the maximal transmittance for all the coatings at 395 nm (inset in Figure 5; see dashed line), when the glass was coated with polyacrylic acid, the transmittance slightly decreased to 96.8%. In the case of functionalized based coatings, 0.1 wt % f-TiO_2_–SiO_2_/PAA and 0.5 wt % f-TiO_2_–SiO_2_/PAA, the maximal transmittances decreased to 96.4% and 95.8%, respectively. Coatings with non-functionalized nanoparticles showed the lowest maximal transmittances of 95.4% for 0.1 wt % TiO_2_–SiO_2_/PAA and 94.5% for 0.1 wt % f-TiO_2_–SiO_2_/PAA. This behavior could be attributed to the presence of SiO_2_ nanoparticles; it has been reported that SiO_2_ nanoparticles increase the transmittance percentage in binary film systems of TiO_2_–SiO_2_ [7] as a result of their low refractive index. Therefore, SiO_2_ nanoparticles can act as an effective antireflection material. By looking at the inset in Figure 5, it can be distinguished that the nanocomposite coatings showed less than a 2.5% loss in transmittance. Furthermore, there was no shifting in the position of maximal transmittance to longer wavelengths, a clear signal of an even thickness and insignificant scattering effect caused by the nanoparticles. On the basis of these results, the nanocomposite coatings obtained did not considerably reduce the transmittance of the glass; this is an interesting property for optical applications in which high transmittance is usually required.

### 3.4. Morphology and Roughness Analysis of f-TiO_2_–SiO_2_ Nanocomposite Coatings

**Figure 6:**
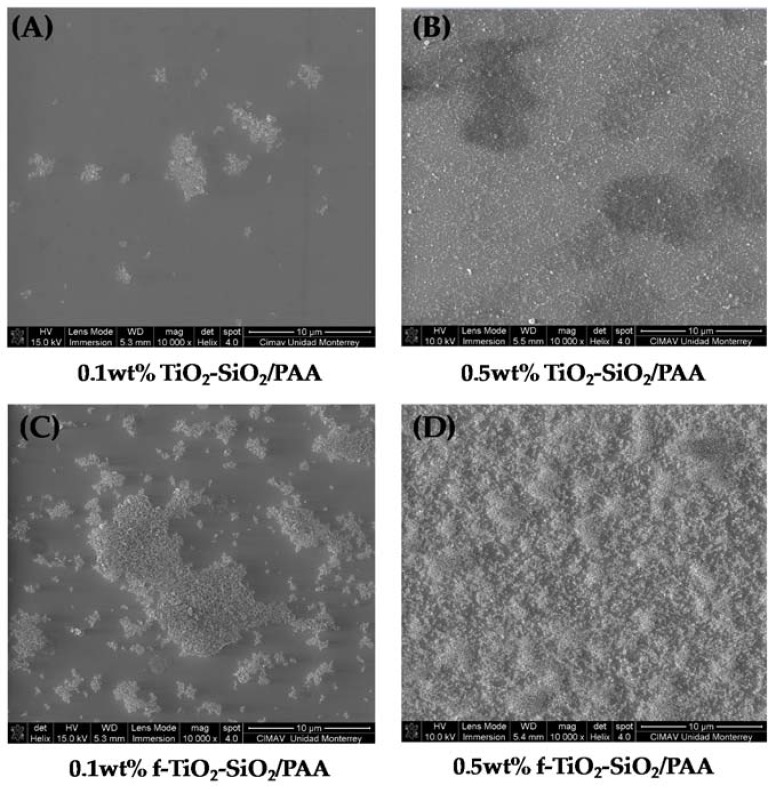
Scanning electron microscopy (SEM) micrographs of nanocomposite coatings: (**A**) 0.1 wt % TiO_2_–SiO_2_/PAA, (**B**) 0.5 wt % TiO_2_–SiO_2_/PAA, (**C**) 0.1 wt % f-TiO_2_–SiO_2_/PAA, and (**D**) 0.5 wt % f-TiO_2_–SiO_2_/PAA.

Figure 6 shows the top-view images of all samples by SEM. In the micrographs is observed that the nanocomposite 0.1 wt % f-TiO_2_–SiO_2_/PAA (Figure 6C) and 0.5 wt % f-TiO_2_–SiO_2_/PAA (Figure 6D) coatings showed a better dispersion than the 0.1 wt % TiO_2_–SiO_2_/PAA (Figure 6A) and 0.5 wt % TiO_2_–SiO_2_/PAA (Figure 6B) samples. The coatings with the functionalized nanoparticles were uniform, and small agglomerations of TiO_2_–SiO_2_ particles were distributed randomly on the surface. Figure 6A–D also indicates that an increase in the TiO_2_–SiO_2_ concentration increased the coverage of nanoparticles on the surface. This grade of dispersion created roughness on the top surface; this characteristic was more evident for coatings with functionalized nanoparticles.

**Figure 7:**
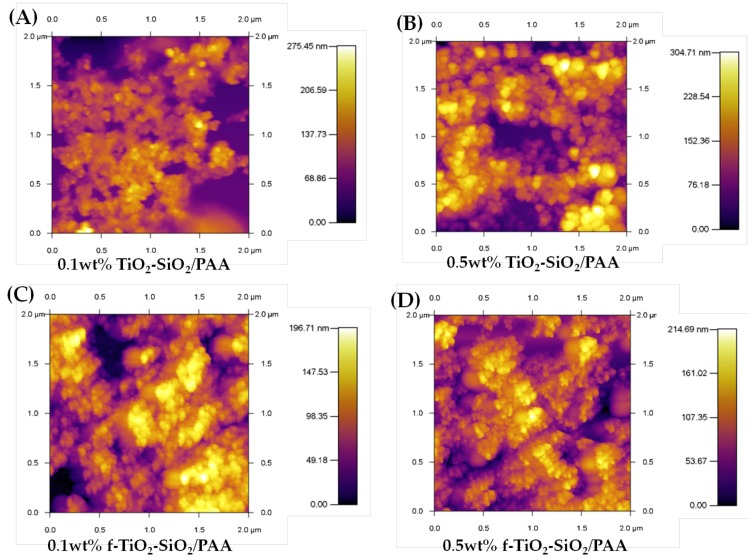
Atomic force microscopy (AFM) images of nanocomposite coatings: (**A**) 0.1 wt % TiO_2_–SiO_2_/PAA, (**B**) 0.5 wt % TiO_2_–SiO_2_ /PAA, (**C**) 0.1 wt % f-TiO_2_–SiO_2_/PAA, and (**D**) 0.5 wt % f-TiO_2_–SiO_2_/PAA.

Contact-mode AFM images of 2 µm × 2 µm for all the nanocomposite coatings are shown in Figure 7. The nanocomposite coating of 0.5 wt % f-TiO_2_–SiO_2_/PAA (Figure 7D) showed agglomerates, where evidently SiO_2_ nanoparticles surrounded TiO_2_ nanoparticles; this behavior was also observed in the nanocomposite coating for 0.1 wt % f-TiO_2_–SiO_2_/PAA.

**Figure 8:**
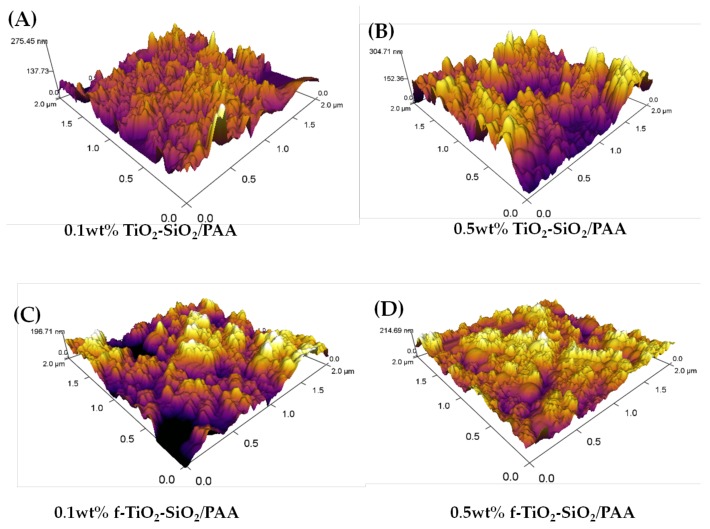
Three-dimensional atomic force microscopy (AFM) images of nanocomposite coatings: (**A**) 0.1 wt % TiO_2_–SiO_2_/PAA, (**B**) 0.5 wt % TiO_2_–SiO_2_/PAA, (**C**) 0.1 wt % f-TiO_2_–SiO_2_/PAA, and (**D**) 0.5 wt % f-TiO_2_–SiO_2_/PAA.

The change in surface roughness was confirmed by AFM measurements. The root-mean-squared roughness (*R*_rms_) revealed that the nanocomposite coatings of 0.1 wt % TiO_2_–SiO_2_/PAA (Figure 8A), 0.5 wt % TiO_2_–SiO_2_/PAA (Figure 8B), 0.1 wt % f-TiO_2_–SiO_2_/PAA (Figure 8C), and 0.5 wt % f-TiO_2_–SiO_2_/PAA (Figure 8D) presented roughnesses of 37.50, 54.88, 18.01, and 25.69 nm, respectively. When the roughness data and water contact angles were correlated, it could be observed that the roughnesses for 0.1 wt % TiO_2_–SiO_2_/PAA, 0.5 wt % TiO_2_–SiO_2_/PAA, and 0.1 wt % f-TiO_2_–SiO_2_/PAA produced hydrophilic coatings with contact angles of 32°, 17°, and 13°, respectively, while that for 0.5 wt % f-TiO_2_–SiO_2_/PAA resulted in a superhydrophilic coating with contact angles of <5°. Therefore, the decrease in the water contact angle could be attributed to the surface roughness, in agreement with the model discussed later.

## 4. Discussion

On the basis of the previous results, it can be seen that nanoparticle functionalization plays an important role in the properties and performance of nanocomposites as functional coatings. In order to understand the interactions between the constituents in the present system, giving the resultant functional properties, further discussion is needed.

In general, the wettability of a solid surface with a liquid is governed both by its chemical composition and by its microstructure (or surface roughness). The Wenzel [25] and Cassie–Baxter [26] models are two well-known wetting models commonly used to correlate the contact angle with surface roughness. To explain our results, the Wenzel model is considered.

According to the Wenzel model, the apparent contact angle on a rough surface, *θ_W_*, is expressed as

(1) $$\cos\theta_{W}=r\left(\cos\theta \right),$$

where *θ* is the contact angle on the flat surface and *r* is the roughness factor, defined as the ratio of the true area of the solid surface to its projection area. Because *r* is always larger than 1, from Equation (1), *θ_W_* is less than *θ* if the surface is originally hydrophilic (*θ* < 90°).

This means that the hydrophilic properties are enhanced when the roughness of the hydrophilic surface is increased.

From Figure 7 and Figure 8, it can be seen that the surface was quite rough, which partially explains the wettability of the coating. However, to reach such roughness, the state of the nanoparticle dispersion is an important issue. This is where the functionalization of the TiO_2_–SiO_2_ nanoparticles makes a contribution to the superhydrophilicity and comes into consideration. As seen in Section 3.1, an effect was achieved with the nanocomposite that pointed to the functionalization of the nanoparticles as suggested by the XPS spectra (cf. Figure 1). To explain the functionalization in the next section, we consider sonication as a critical step.

As mentioned in the methodology, ultrasonication was an approach for nanoparticle functionalization; an energy of 594 kJ/g was used with the purpose to achieve functionalization and assist dispersion. As a result of their extremely large surface-area/particle-size ratio, nanoparticles tend to strongly agglomerate, hence reducing the resultant expected properties of the nanocomposite.

The interactions that normally occur in ultrasonic cavitation with compounds are complex [27]; alone, they usually have sufficient energy for organic compound degradation [28] or for catalysis of more complex reactions in sonocatalysis [29]. Where normally reactions do not proceed without sonication, sonication enables other paths for the reaction. Along with the system, there is the presence of metallic oxide nanoparticles, which usually contain, depending on their synthesis or further treatment, surface hydroxyl groups, which are often energetically difficult to remove [30,31].

**Figure 9:**
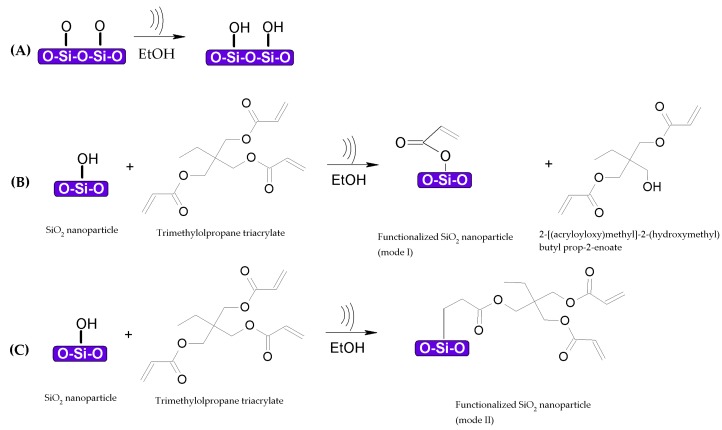
Proposed reaction mechanism for SiO_2_ nanoparticles functionalized with trimethylolpropane triacrylate (TMPTA) under sonication conditions. (**A**) hydroxyl groups are attached to SiO_2_ nanoparticles after the first step, addition of TMPTA results in (**B**) functionalized SiO_2_ nanoparticles with an acrylic surface moiety (mode I) and (**C**) functionalized SiO_2_ nanoparticles with a vinyl group (mode II).

It is known that the reactivity of a substance increases with smaller particles. In this study, SiO_2_ has a specific surface area of 200 ± 25 m^2^/g, while that of TiO_2_ is 90 ± 20 m^2^/g (according to the supplier), meaning that SiO_2_ has a better affinity with adsorption and reaction in the system. It is also known that SiO_2_ can react with trialkoxyalkyl molecules at high temperatures to form an ester between silanol groups and ester groups [11]. The sonication process provides the required energy for the reaction to proceed, as shown in Figure 9A. Thus, one product of SiO_2_ and TMPTA is an acrylic surface moiety (mode I), as seen in Figure 9B; the other pathway produces SiO_2_ functionalized via a vinyl group (mode II), as shown in Figure 9C. This is in accordance with the XPS Si2p spectra, where evidence of Si–C bonding is present. The proposed reaction of this process is shown in Figure 9.

**Figure 10:**
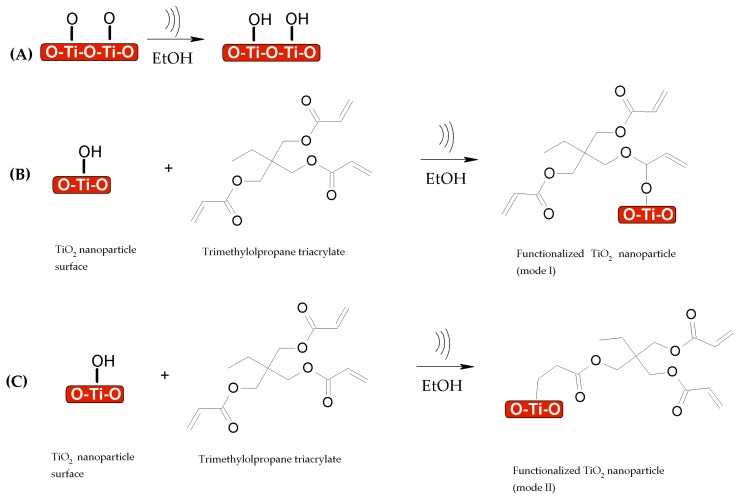
Proposed reaction mechanism for TiO_2_ nanoparticles functionalized with trimethylolpropane triacrylate (TMPTA) under sonication conditions. (**A**) hydroxyl groups are attached to TiO_2_ nanoparticles after the first step, addition of TMPTA results in (**B**) functionalized TiO_2_ nanoparticles (mode I) and (**C**) functionalized TiO_2_ nanoparticles with a vinyl group (mode II).

For TiO_2_ nanoparticles, it is known that titanium can bind to acrylic systems via dentate ligands [13]. This could explain the slight decrease in the number of carbonyl groups in the TMPTA, suggesting a more complex interaction in the cavitation process in order to be reduced. It is known that excited TiO_2_ generates electrons in the conduction band, which can be transferred to organic compounds [32]. Additionally, there is evidence that TiO_2_ can undergo the same phenomena under ultrasonication conditions [33,34]; thus the reduction of carbonyl groups could also be suggested. The reaction between TiO_2_ and TMPTA to functionalize TiO_2_ is shown in Figure 10. It occurs in a similar manner as in the case of SiO_2_ nanoparticles, for which the sonication process makes the particles more reactive, as shown in Figure 10A. It then follows two suggested routes, one in which the carbonyl group can be reduced by the sonication process and TiO_2_ interacts as is shown in Figure 10B, obtaining the functionalized TiO_2_ (mode I), and the second in which TiO_2_ would be bonded by vinyl interactions in TMPTA (mode II), as is shown in Figure 10C.

For the functionalization of the binary system, it can be concluded that it should consist of a combination of both phenomena. For the binary (TiO_2_–SiO_2_) system, both metallic oxides are functionalized at the same time, meaning that in parallel with the cavitations, enough energy is exerted for nanoparticles to collide during the functionalization. As a function of concentration, higher concentrations represent a higher collision frequency, as studied by Pradhan [35]. Additionally, TiO_2_ and SiO_2_ have different sizes, resulting in a difference in surface energy; the system nature is to decrease this excess of surface energy, and thus the particles tend to form preferential arrangements between each other. Thus, the particles are functionalized under these conditions.

**Figure 11:**
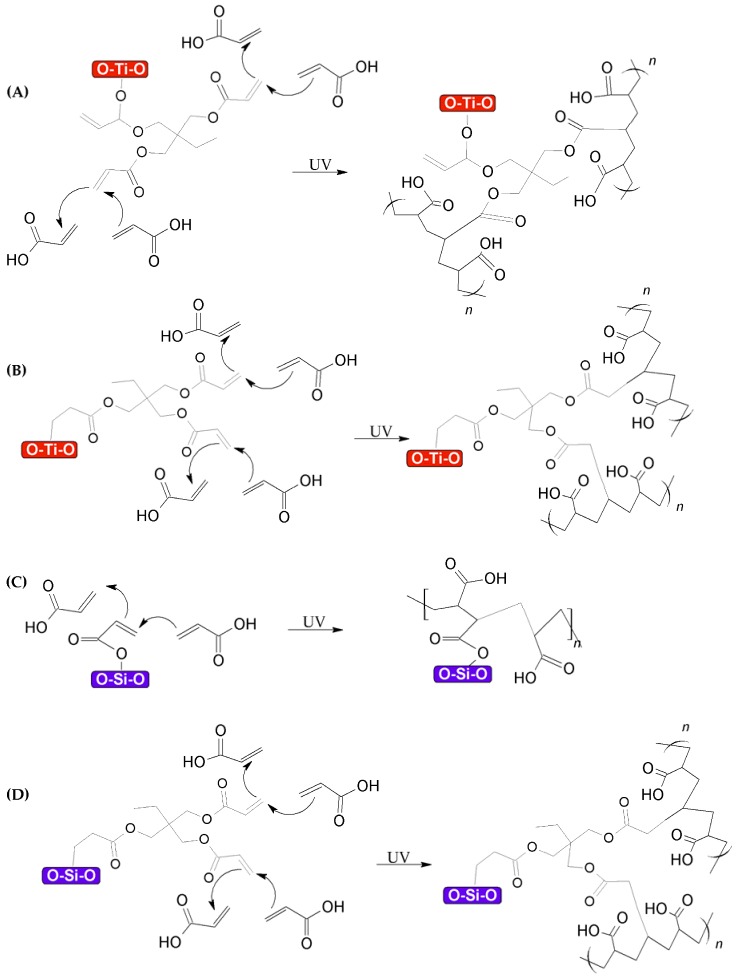
Proposed reaction mechanism under UV crosslinking conditions of acrylic acid in presence of (**A**) functionalized TiO_2_ nanoparticles (mode I), (**B**) functionalized TiO_2_ nanoparticles (mode II), (**C**) functionalized SiO_2_ nanoparticles (mode I), and (**D**) functionalized SiO_2_ nanoparticles (mode II).

Additionally, the superhydrophilicity of the present system could be attributed to the synergy between the f-TiO_2_–SiO_2_ nanoparticles and the acrylic matrix. TMPTA functionalization of the nanoparticle surface showed an intact acryloyl group, meaning that its property to propagate through photoinitiation and polymerize via crosslinking remained as is shown in Figure 11. The proposed mechanism shows the way that both modes (I and II) of functionalized TiO_2_ and SiO_2_ nanoparticles could interact in the polymerization under UV conditions (Figure 11A–D).

**Figure 12:**
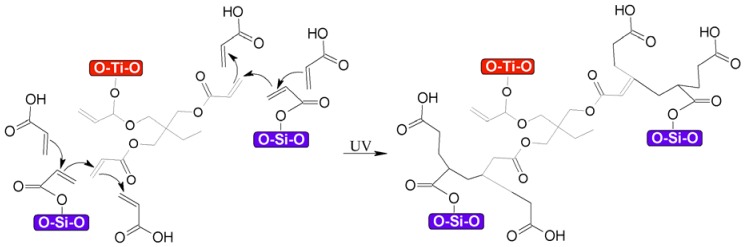
Proposal for the polymerization of moieties between functionalized TiO_2_–SiO_2_ nanoparticles.

**Figure 13:**
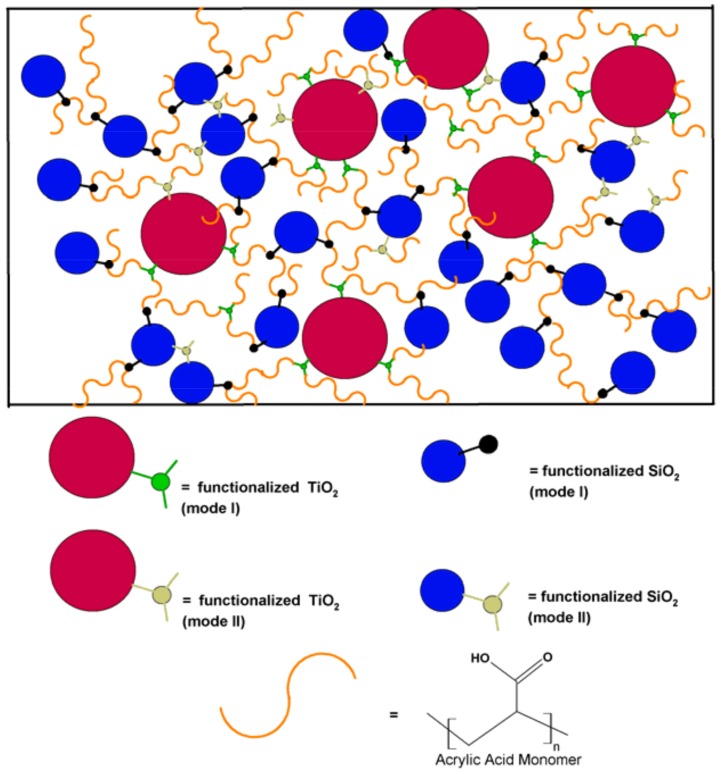
Schematic representation of f-TiO_2_–SiO_2_/PAA nanocomposite, for which both modes of functionalized TiO_2_ and functionalized SiO_2_ are polymerized with acrylic acid monomer, as well as entanglement between functionalized nanoparticles.

From the crosslinking and polymerization of both functionalized nanoparticles shown in Figure 11, it can be concluded that the binary system should be a mixture of both reactions, as represented in Figure 12, whereby the polymerization between vinyl groups on the surface of the nanoparticles can also take place. For clarity purpose, a schematic representation of the final nanocomposite appears in Figure 13.

## 5. Conclusions

A facile method for surface functionalization of a TiO_2_–SiO_2_ binary system was achieved using TMPTA with the aid of ultrasonication. Covalently functionalized TiO_2_–SiO_2_ nanoparticles were dispersed in an acrylic matrix, resulting in the formation of nanocomposite coatings with hydrophilic and superhydrophilic behavior. These properties were enhanced without sacrificing visible light transmission. Superhydrophilic coatings with water contact angles below 5° have many important applications, such as for self-cleaning coatings. These results provide valuable guidance for the design and manufacture of self-cleaning coatings.

## 6. Patents

This study generated the Patent Request No. MX/a/2016/014953 at the Mexican Institute of Industrial Property.

## Figures and Tables

**Table 1:** Nomenclature for nanocomposite coatings.

Nanocomposite	Nanoparticle	wt %	Nomenclature
TiO_2_–SiO_2_/PAA	Non-functionalized	0.1	0.1 wt % TiO_2_–SiO_2_/PAA
		0.5	0.5 wt % TiO_2_–SiO_2_/PAA
f-TiO_2_–SiO_2_/PAA	Functionalized	0.1	0.1 wt % f-TiO_2_–SiO_2_/PAA
		0.5	0.5 wt % f-TiO_2_–SiO_2_ /PAA
